# Supplementary material for: Convergent depression of activity-dependent bulk endocytosis in rodent models of autism spectrum disorder
Source: Mol Autism. 2025 Apr 16;16:26. doi: 10.1186/s13229-025-00660-6 (PMC12004638; doi:10.1186/s13229-025-00660-6)
Supplement: Supplementary file 1 — Supplementary Material 1 [file 13229_2025_660_MOESM1_ESM.pdf]

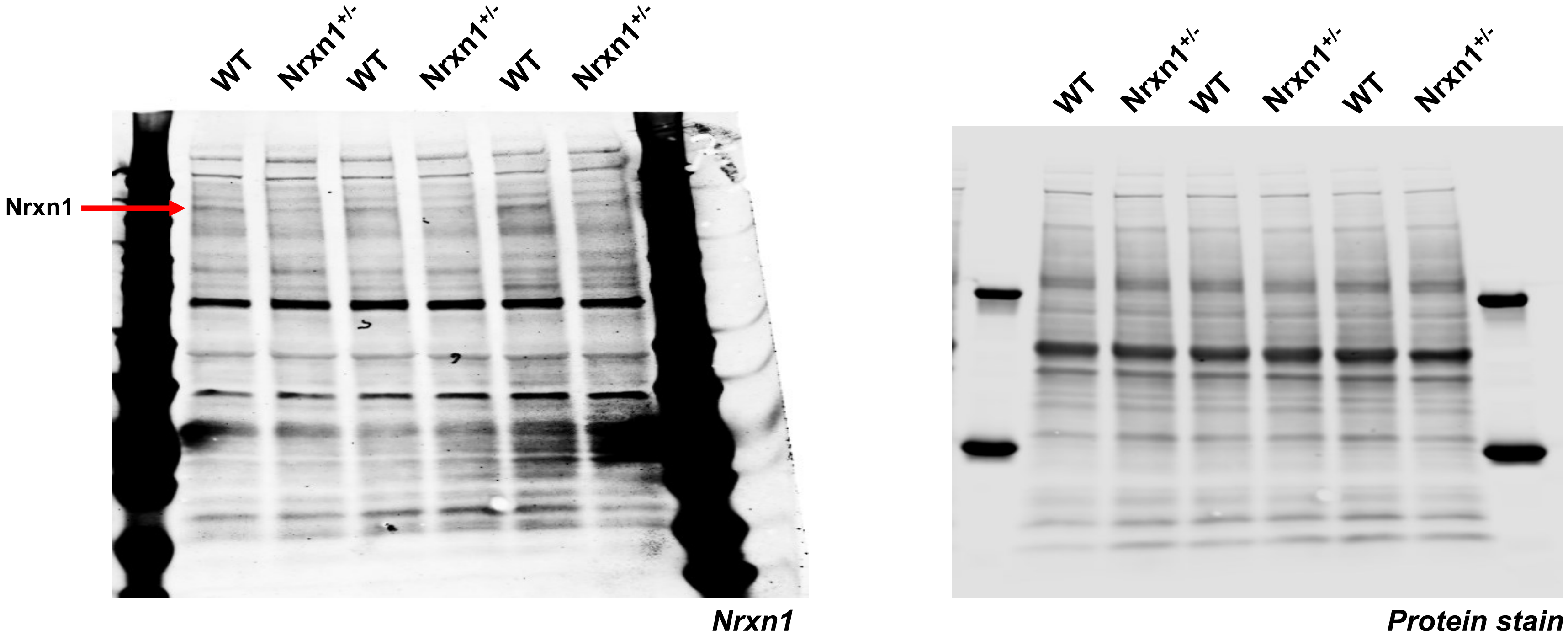

Raw blots from Figure 2A,B) - Hippocampal synaptosome lysates from either wild-type (WT) or *Nrxn1*$^{+/-}$ rats were probed for the presence of Neurexin-1 (Nrxn1, left) or total protein (right). In the Nrxn1 blot, red arrow indicates the 160 kDa band that changes in *Nrxn1*$^{+/-}$ synaptosomes.

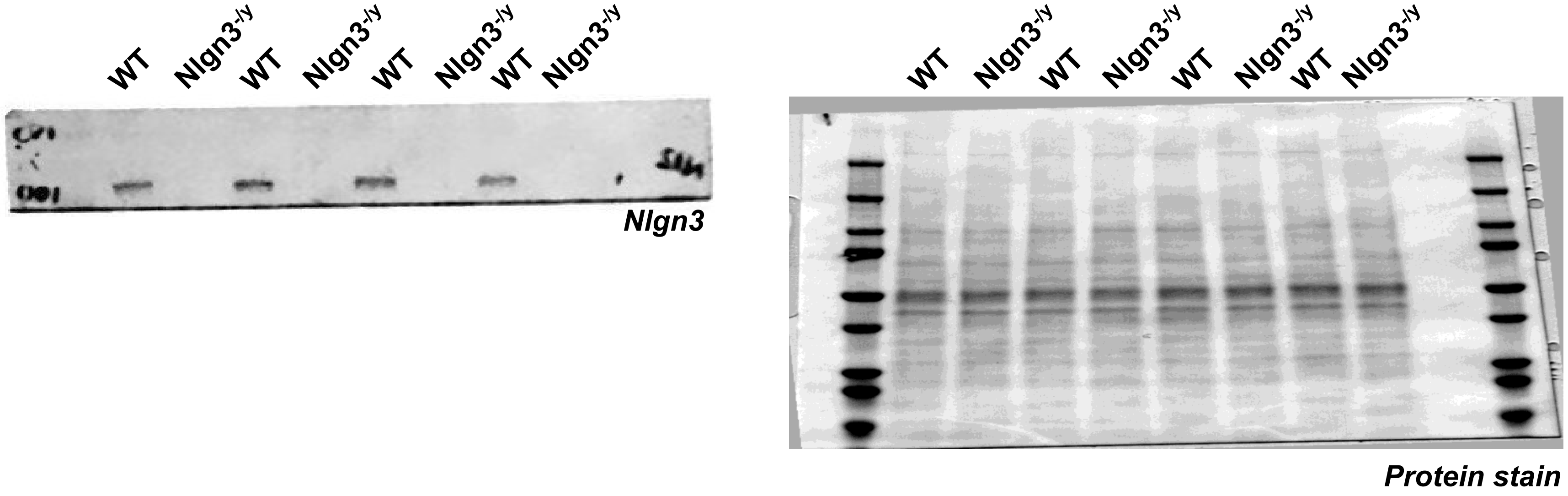

Raw blots from Figure 3A,B) - Hippocampal synaptosome lysates from either wild-type (WT) or *Nlgn3*$^{-/y}$ rats were probed for the presence of Neuroligin-3(Nlgn3, left) or total protein (right). The Nlgn3 blot was cut before probing with antibody, due to the fact that the remainder of the membrane was being probed for a different protein.

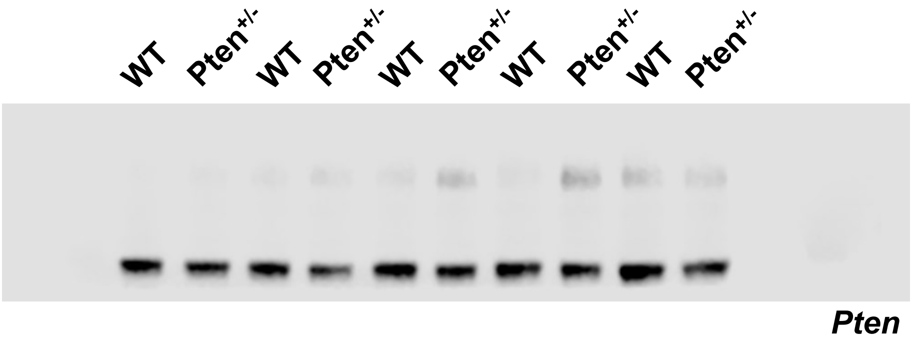

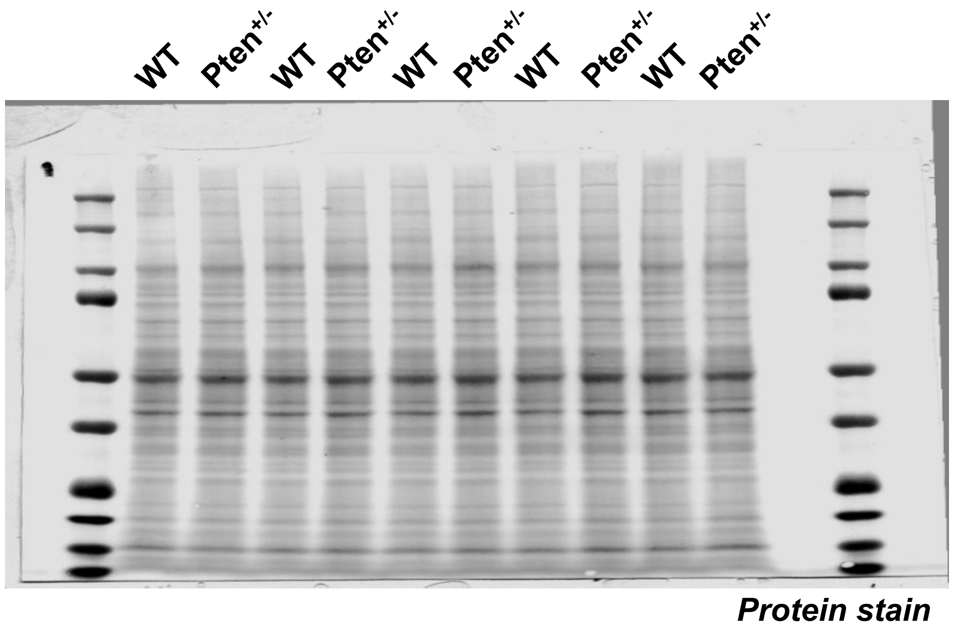

Raw blots from Figure 6A,B) - Hippocampal synaptosome lysates from either wild-type (WT) or *Pten*$^{+/-}$ rats were probed for the presence of Pten (left) or total protein (right). The Pten blot was cut before probing with antibody, due to the fact that the remainder of the membrane was being probed for a different protein.
